# Hydrothermal preparation of silver telluride nanostructures and photo-catalytic investigation in degradation of toxic dyes

**DOI:** 10.1038/srep20060

**Published:** 2016-01-25

**Authors:** Sousan Gholamrezaei, Masoud Salavati-Niasari, Davood Ghanbari, Samira Bagheri

**Affiliations:** 1 Institute of Nano Science and Nano Technology, University of Kashan, Kashan, P.O. Box 87317-51167, I. R. Iran; 2 Nanotechnology & Catalysis Research Centre (NANOCAT), IPS Building, University of Malaya, 50603 Kuala Lumpur, Malaysia

## Abstract

Different morphologies of Ag_2_Te nanostructures were synthesized using TeCl_4_ as a new precursor and hydrazine hydrate as reducing agent by a hydrothermal method. Various parameters that affect on morphology and purity of nanostructures were optimized. According to our experiments the best time and temperature for preparation of this nanostructure are 12 h and 120 °C. The photo-catalytic behaviour of nanostructures in presence of UV- visible light for degradation of methyl orange was investigated. Results show that the presence of UV light is necessary for an efficient degradation of dye in aqueous solution. On the other hand, as observations propose the Ag_2_Te reveal a strong photoluminescence peak at room temperature that could be attributed to high level transition in the semiconductor. Nanostructures were characterized by X-ray diffraction (XRD), scanning electron microscopy (SEM), Fourier transform infrared (FT-IR) techniques and UV–visible scanning spectrometer (UV-Vis).

Effluent treatment, pollution decomposition and energy shortage are commonly crucial problems for humanity^1^. Wastewater treatment and degradation of liquid pollutant can be an essential concern for the whole of society due to the enormous circulation and mobility of water systems in the environment^2^. Wastewater treatment has influence on human health and hygiene. On the other hand, it has a large influence on the whole ecosystem for every creatures. Therefore, an environmental friendly and low cost technique for separation of impurities from water sources is necessary^3^. Generally, degradation of pollution from effluent manufacturing, including aromatic compounds and organic dyes, introduces a potential hazard to the environment that is required to distribute before discharging effluent to environment^4^.

Methyl orange (MO) is an essential case of azo dye and it has extensively application as a coloring agent in different industries such as textile, food, leather and pharmaceutical. MO can use as coloring agent for the recognition of hydrogen gas and determination of Itopride hydrochloride^5^,^6^. MO is toxic, mutagenic and carcinogenic and discharge of MO from dye manufacturing into water bodies that makes a lot of health hazards^7^,^8^,^9^. MO cannot degrade biologically; therefore special methods are necessary for its degradation. Various methods such as physical methods (coagulation, reverse osmosis, membrane filtration), chemical approach (reduction, oxidation, ion exchange, complex metric methods) and biological techniques (aerobic, anaerobic) are used for degradation of MO^10^. In recent times, advanced oxidation processes (AOP) for removal of organic pollutants from water are attracted a lot of attention^11^,^12^,^13^,^14^. These procedures involve the treatment of wastewater by *in situ* generation of hydroxyl radicals which have strong oxidizing agents and can oxidize pollutant to CO_2_ and H_2_O^15^. This process is very fast and does not generate any solid waste. These procedures are performed in presence of ozone (O_3_), hydrogen peroxide (H_2_O_2_) and/or UV light. In these reactions, semiconductor materials can act as catalyst for the degradation of pollutant^16^.

Silver telluride (Ag_2_Te) is a significant semiconductor (A_2_^I^B^IV^ group) with a hopeful thermoelectric properties, large magneto resistance and high electrical conductivity^17^,^18^ that has a large wide application in nonlinear optical devices, ion selective electrode, electrochemical storage cells, infrared sensors, solar cell and biological sensors^19^,^20^,^21^,^22^,^23^. Recently, a lot of attention focused on new type nano-devices based on Ag_2_Te nanostructures which are very importance because of their behavior that are differed from corresponding polycrystalline bulk in mechanical, electrical and magnetic properties^24^,^25^,^26^. Up to now, a number of techniques for the synthesis of one-dimensional (1D) Ag_2_Te nanostructures are investigated. Herein, we used a hydrothermal method to synthesis this semiconductor nanostructures in a mild condition. The hydrothermal approach is selected because of its low cost, high efficiency and potentiality for mass-production^27^,^28^,^29^. In view of the fact the uniqueness of nanostructures largely depends on their dimension size and shape. Hydrothermal is an exclusive process for preparation of nanostructures with specific and controlled shape, while other techniques such as sol-gel and sonochemical give mostly nanoparticle morphology. The hydrothermal method supplies suitable morphology orientation^30^. In different special conditions; e.g., high temperature and pressure, these approaches produce different shapes *in situ* and shape several morphologies such as nanoparticles, nanorods and nanoplates were obtained^31^. Our method for synthesis of Ag_2_Te is very simple, low cost and can be scale up that nontoxic precursors and solvent were used (TeCl_4_ as Te precursor).

To our acknowledgment, it is the first time that TeCl_4_ was used to prepare Ag_2_Te nanostructures. TeCl_4_ can produce TeO_3_^2−^ in solution but in comparison with Na_2_TeO_3_ the reactivity and the rate of preparation TeO_3_^2−^ via TeCl_4_ is very large. Since TeCl_4_ resulted in the increasing the rate of nucleation, smaller nanostructures were obtained^32^,^33^,^34^.

## Results and Discussion

### Optimization of Ag_2_Te nanostructures

**Figure 1:**
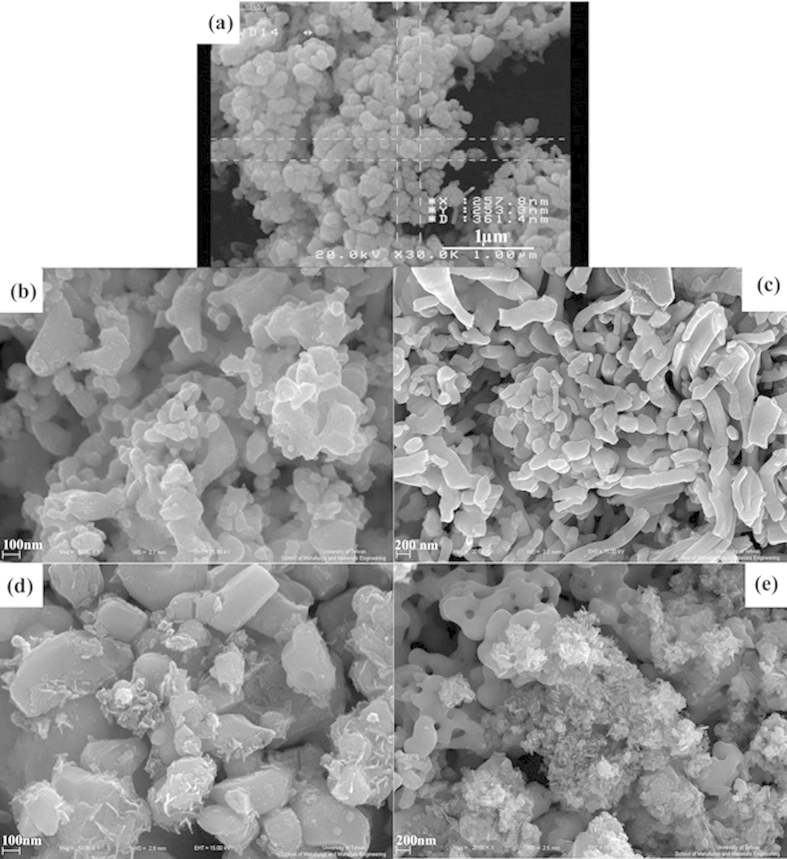
SEM images of Sample No. (**a**) 1 (**b**) 2 (**c**) 3 (**d**) 4 (**e**) 5.

**Figure 2:**
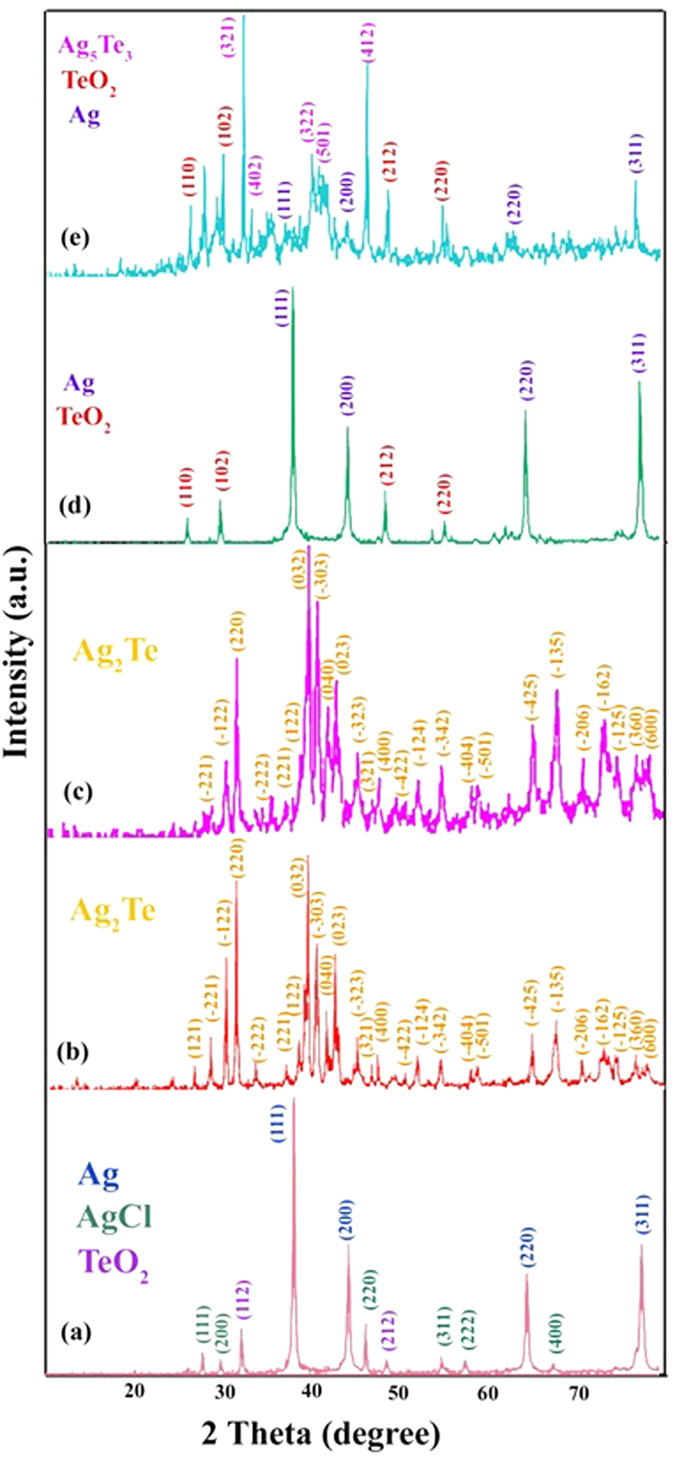
XRD patterns of sample No. (**a**) 1 (**b**) 2 (**c**) 6 (**d**) 10 (**e**) 11.

During the preparation of Ag_2_Te nanostructures, different condition such as fabrication of Ag_2_Te by co-precipitate method in the room temperature was investigated. A gray precipitate was formed by adding the reducing agent to the mixed solution of Ag and Te ion. SEM image of the gray precipitate is shown in Fig. 1a. According to the image, precipitates were formed from spherical particles with average 250–300 nm diameter. The XRD pattern of spherical particles is shown in Fig. 2a. According to the XRD pattern the product is a mixture of Ag, AgCl and TeO_2_ powder, consequently the Ag_2_Te cannot be formed in room temperature and ambient pressure. Because of mentioned reason, the hydrothermal method was selected for preparation of Ag_2_Te nanosturctures and the effective parameters on morphology and purity of product such as time, temperature, reducing agent and presence of different surfactant were studied.

### The effect of time and temperature

Figure 2b illustrates XRD pattern of sample No. 2 that was prepared in 6 h and 160 °C. The pattern of the as-prepared Ag_2_Te nanostructures were indexed as a pure monoclinic phase (space group: P2/n), which are very close to the literature values (JCPDS No. 34–0142), the narrow sharp peaks indicate that the Ag_2_Te nanoparticles are well crystallized. The crystallite size measurements are also calculated by Scherrer equation, Dc = Kλ/βcosθ, where β is the width of the observed diffraction line at its half intensity maximum, K is the so-called shape factor, which usually takes a value of about 0.9, and λ is the wavelength of X-ray source used in XRD. The estimated crystallite size of sample No. 2 is 47 nm.

Figure 1b–e shows SEM images of sample No. 2–5 which were prepared in different times from 6 h to 24 h in constant temperature at 160 °C. The morphology of product were changed by increasing the reaction time, in Fig. 1b synthesized Ag_2_Te does not have a unique shape by increasing the reaction time to 12 h. By increasing the reaction time to 18 h and 24 h the products were arrayed in semi-rode nanostructures. So, the best time for preparation of Ag_2_Te was 12 h.

**Figure 3:**
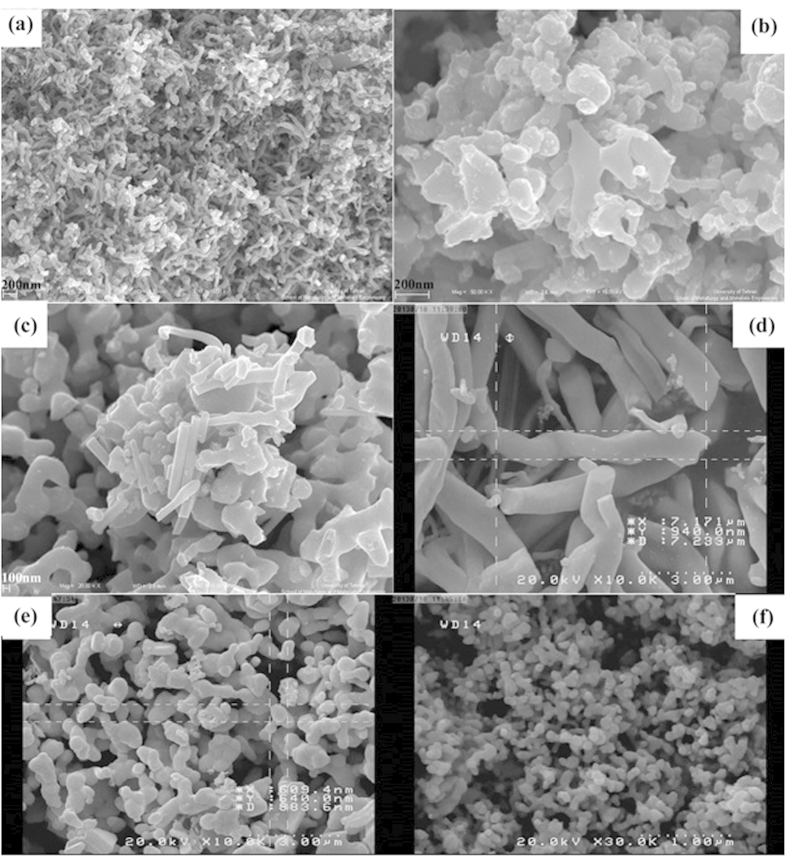
SEM images of Sample No. (**a**) 6 (**b**) 7 (**c**) 8 (**d**) 9 (**e**) 10 (**f**) 11.

Figure 3a shows the SEM images of sample No. 6–9 in constant time (12 h) and various temperatures. In 120 °C the product is made up from many short nanorod. By increasing the temperature to 200 °C aggregated nanostructures were formed.

The XRD pattern of sample No. 6 which was synthesized in 120 °C and 12 h is shown in Fig. 2c. The estimated crystallite size of sample No. 6 is 35 nm. According to experiments the optimum of time and temperature for preparation of nano-rods Ag_2_Te nanostructures are 12 h and 120 °C.[Fig f4]

Figure 5 shows Transmission electron microscope, HRTEM and SAED of as prepared Ag_2_Te (Sample No. 6). According to these images, Ag_2_Te were made up capsular nanostructures with about 31 nm lengths and about 20 nm widths. HRTEM and SAED patterns show that product was formed from poly crystalline nanostructures. The distance of crystalline plate nanostructures was 0.82 and 0.34 nm that was confirmed with the XRD patterns.

**Figure 4 f4:**
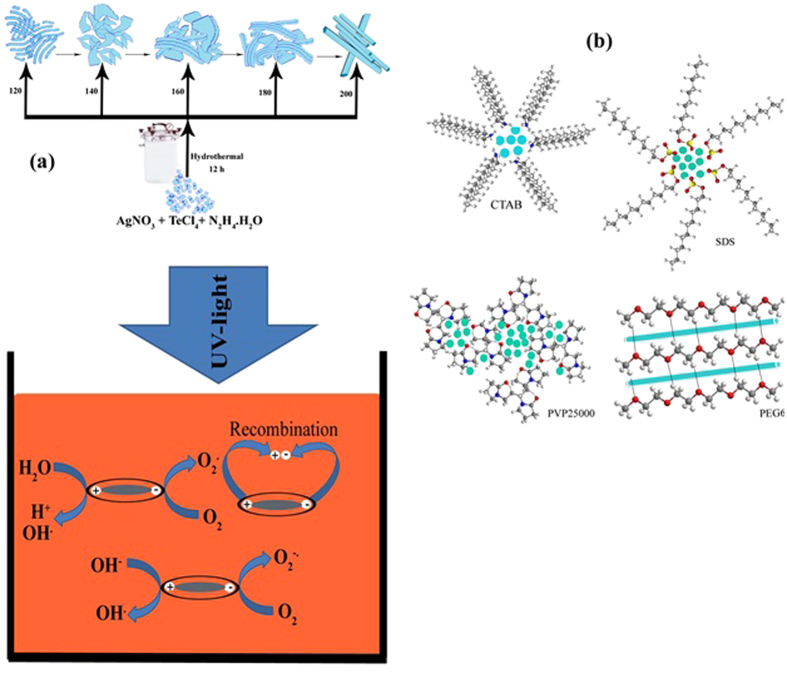
(**a**) Schematically mechanism of formation Ag_2_Te nanostructures in different temperature (**b**) Schematically arrays of different surfactant on solution for preparation of Ag_2_Te nanostructures (**c**) Schematically mechanism of photocatalytic degradation of MO under UV irradiation by Ag_2_Te nanostructures (Sample No. 6).

**Figure 5 f5:**
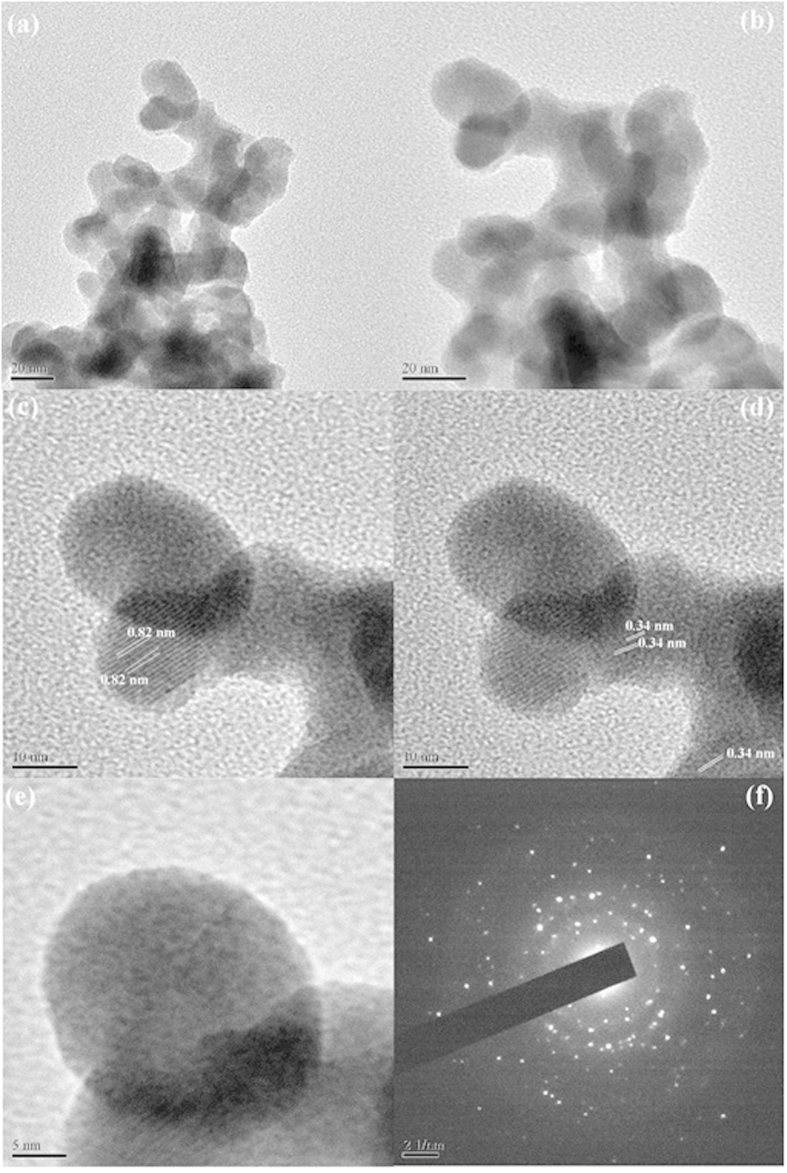
(**a**,**b**) TEM image (**c**–**e**) HRTEM image (**f**) SAED pattern of Sample No. 6.

Figure 4a schematically illustrates mechanism for preparation of Ag_2_Te nanostructures in different temperatures. In accordance with[Fig f5]Ostwald ripening by increasing in temperature the primary nanostructures grow and micro-rod structures was formed.

### The effect of reducing agent

Hydrazine monohydrate is a reducing agent with middle power that leads to nanorods of Ag_2_Te. The effect of reducing agent on purity and morphology of Ag_2_Te was investigated in presence of Na_2_SO_3_ as a weaker and KBH_4_ as a stronger reducing agent in versus of N_2_H_4_.H_2_O.

Figure 2d illustrates XRD pattern of synthesized in attendance of various reducing agents. In presence of Na_2_SO_3_ the product was Ag and TeO_2_ nanoparticles; whereas when KBH_4_ were used as reducing agent, in addition to Ag and TeO_2_, Ag_5_Te_3_ also was produced. Therefore because of high power of KBH_4_ Ag_2_Te were polymerized and formed the Ag_5_Te_3_.

Figure 3e shows SEM image of the mixture of Ag and TeO_2_ which achieved from reaction of AgNO_3_ and TeCl_4_ and Na_2_SO_3_. The average diameter of these nanoparticles is 650 nm. Figure 3f illustrates the SEM image of as-synthesized powder in presence of KBH_4_, the prepared nanoparticles in these conditions have lower diameter than nanoparticles which were prepared in the presence of KBH_4_ (According to the Ostwald ripening, reduction in diameter is due to the higher rate of reaction in presence of KBH_4_).

### The effect of surfactant

The effect of various surfactants on the morphology of Ag_2_Te was studied. Three types of surfactants were used in our experiments:

1. Cationic surfactant
2. Anionic surfactant
3. Polymeric surfactants

**Figure 6:**
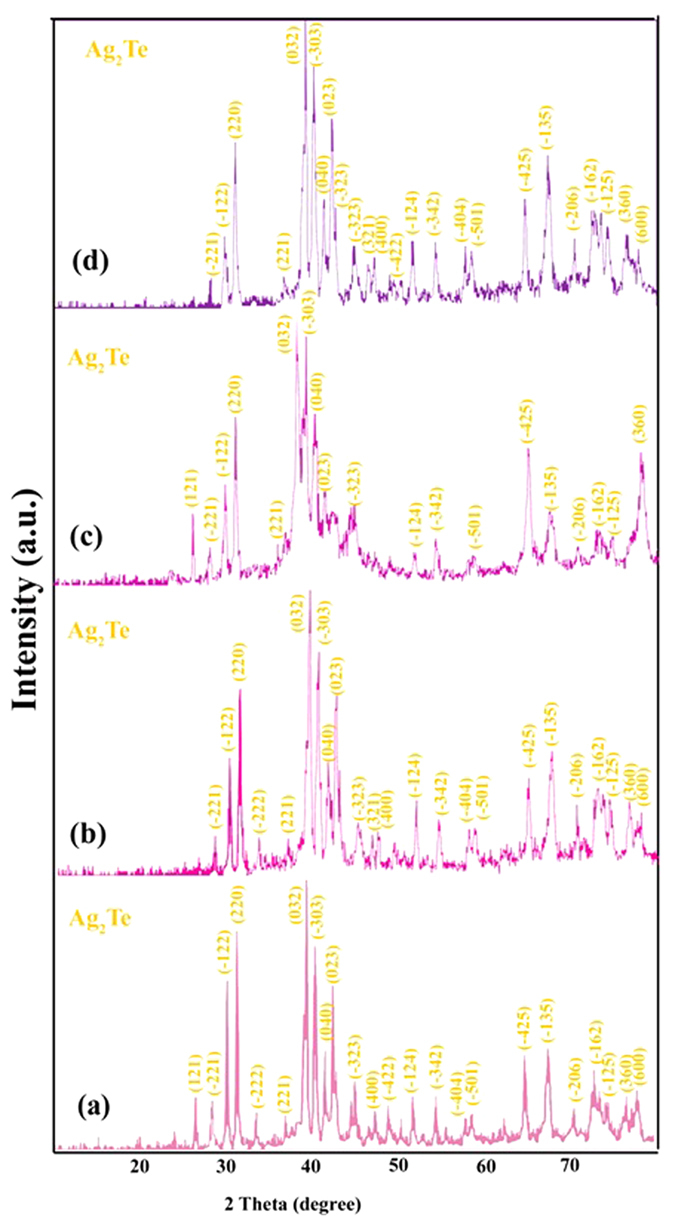
XRD patterns of sample No. (**a**) 13 (**b**) 15 (**c**) 18 (d) 20.

Figure 6 illustrates XRD patterns of as-synthesized Ag_2_Te nanostructures in presence of CTAB (Fig. 6a), SDS (Fig. 6b), PVP25000 (Fig. 6c) and PEG600 (Fig. 6d). In attendance all of the materials, Ag_2_Te were produced as product of reaction. Crystalline sizes of as-synthesized nanostructures were estimated 35, 35, 45 and 46 nm respectively. Consequently, the crystalline phase size of nanostructures in presence of ionic surfactant is lower than the size in the presence of polymeric surfactant. This is due to stronger interaction of ionic surfactant toward polymeric surfactant with primary ions.

**Figure 7:**
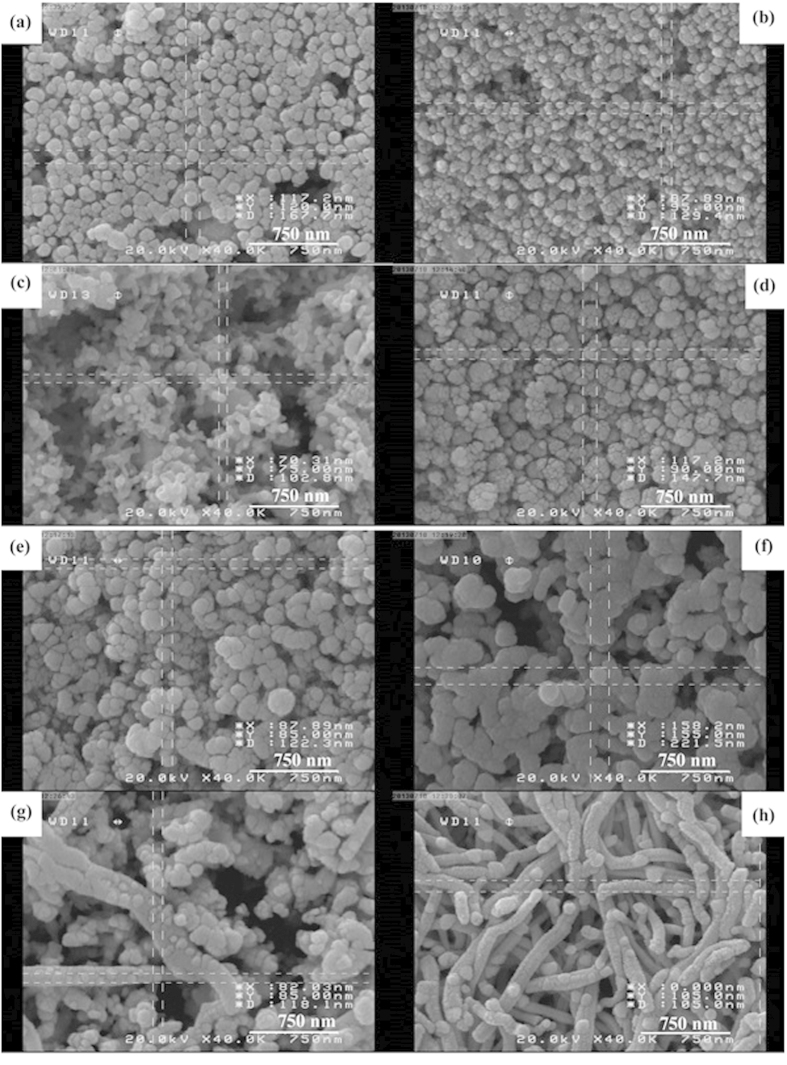
SEM images of Sample No. (**a**) 12 (**b**) 13 (**c**) 14 (**d**) 15 (**e**) 16 (**f**) 17 (**g**) 18 (**h**) 19.

Figure 7a–d shows SEM images of as-synthesised Ag_2_Te nanostructures in presence of ionic surfactant with different mole ratio of surfactant to precursors. In presence of CTAB as a cationic surfactant (Fig. 7a,b), the morphology of product in different mole ratios were constant but the size of nanostructures were changed. By increasing amount of CTAB the size of as-prepared nanoparticles were decreased. That is due to equalization of positive and negative charge in twice mole ratio of Te: CTAB. Whereas in the presence of SDS as shown in Fig. 7c,d, smaller nanoparticles were achieved in equal mole ratio of Te: SDS. In this concentration the amount of charges in Ag ion and SDS were equal.

Based on researches like Brinker *et al*. it seems the CTAB as surfactant has suitable influence on self-assembly of product. CTAB in aqueous solution was separated to cationic and anionic components. The tellurium ion could be capped the in solution by cationic component and controlled the rate of reaction. In presence of CTAB the morphology of product was nanoparticles. According to concentration of CTAB, the micelles have been formed in solution and these micles have a spherical shapes^35^. Therefore the morphology of final product is nanoparticles.

Figure 7e–h shows the effect of polymeric surfactant on morphology of Ag_2_Te. In the presence of less amount of PVP25000, the nanoparticles have lower size but by increasing the amount of PVP25000, this surfactant destroyed the micelles and the size of as-prepared Ag_2_Te was increased (Fig. 7e,f). But in presence of PEG600 which has lower weight in compared with PVP25000. By increasing the amount of surfactant, morphology of product did not change and it was arrayed in nanorod structures (Fig. 7g,h).

Figure 4 schematically depicts arrays of surfactant in solution. These arrays made various micelles in solution and organized Ag_2_Te nanostructures.

**Figure 8:**
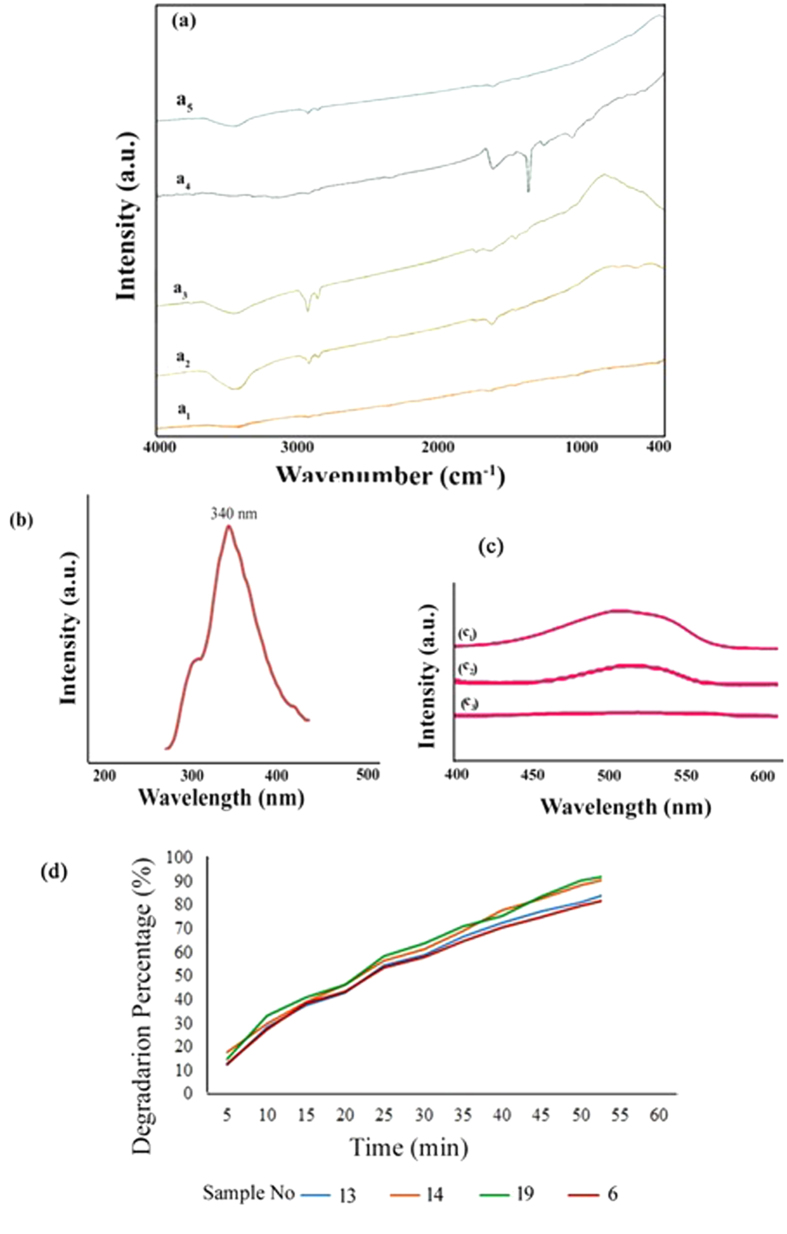
(**a**) FTIR spectra of Sample No. (a_1_) 6 (a_2_) 13 (a_3_) 15 (a_4_) 17 (a_5_) 19 (**b**) PL spectrum and (**c**) UV-Vis spectra of photodegradation of MO with Ag_2_Te as afunction of time (C_1_) 0 min (C_2_) 30 min (C_3_) 60 min of Sample No. 6 (**d**) Photocatalytic degradation digrams.

For investigation purity of Ag_2_Te in presence of different surfactants FT-IR spectroscopy was used. Figure 8a illustrates spectrum of surfactant-free Ag_2_Te. There isn’t any absorption around 400–4000 cm^−1^ that confirmed purity of the product. But in the presence of different surfactants absorption which is responsible to presence of surfactant are shown in FT-IR spectra. The peaks that placed in 3400 and 1640 cm^−1^ in Fig. 8a2–a5 are related to water molecules on the external surface of the sample^36^. Another peak appeared in about 3200 cm^−1^ was appointed to aliphatic CH_2_ in surfactant. In presence of PVP25000 a peak exist in 1400 cm^−1^ that is due to stretching mode of C=O band in PVP.

The room temperature photoluminescence spectrum of Ag_2_Te nanorod which produced in 12 h and 120 °C is shown in Fig. 8b. The rate of scanning was set at 1500 nm/min. The maximum of emission of Ag_2_Te were located in 340 nm (λex = 250 nm). The most general utilize of PL is determination of band gap, or band-to-band transition. The calculated band gap is 3.64 eV which shows a big blue-shifted emission in comparison with bulk samples^37^. The elevated difference between the available band gap values and the measurements can be ascribed to size quantization in the nanocrystalline semiconductor Ag_2_Te. This size quantization occurs because of confinement of electrons and holes in a limited volume of the semiconductor nano-crystallites. The quantum localization effect can be observed on one occasion the diameter of the particle is of the same size as the wavelength of the electron wave function. The electronic and optical properties of small materials deviate substantially from those of bulk materials. A particle performs as if it were free when the limiting dimension is large associated with the wavelength of the particle. Therefore, the band gap stays at its original energy since a continuous energy state. On the other hand, with decreasing the dimension to nanoscale, the energy spectrum turns to distinct. As a result, the band gap shows size dependent and a blue shift was observed in optical illumination as the size of the particles decreases^38^,^39^.

According to other related research works about Te compounds that were reported by our research group^32^,^33^,^40^,^41^,^42^, when TeCl_4_ is used in aqueous solution the TeOCl_2_ and TeO_2_ are formed. The presence of TeO_2_ in solution was approved by XRD pattern of samples No. 1, 10 and 11. On the other hand the AgNO_3_ in presence of TeCl_4_ and resulted HCl (from hydrolysis of TeCl_4_) lead to formation of AgCl. The XRD pattern of sample No. 1 approves the presence and formation of AgCl. The suggested mechanism according to the XRD pattern and related works are shown in as follow:


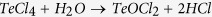



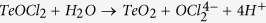



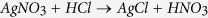



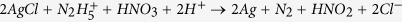



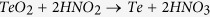



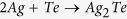


### The photo-catalytic activity

Theoretical calculation show that the band gap of bulk Ag_2_Te semiconductor is very low (around 0.2 eV)^43^,^44^. The photoluminescence study on this compound approved this calculation and show a emission peaks on IR region^45^. By decreasing the size of semiconductor the band gap value was increased. This procedure was observed for Ag_2_Te. The Ag_2_Te nanostructure as other chalcogenides nanostructures have a large bohr exiton radius and the quantum effect could be appropriately observed in these materials^46^,^47^. The photoluminescence emission and calculated band gap for product show an indirect band gap that is equal to 3.46 eV. This band gap is exactly around the band gap of titanium dioxide nanostructure that is the most famous and effective photo-catalyst compound and it seems it is key factor in preparation of photo-catalyst. Therefore the Ag_2_Te has been used as photocatalyst in degradation of azo dye.

The photo-catalytic activity of Ag_2_Te powder was investigated by degradation of MO as an organic pollution. Figure 8c illustrates the UV-Vis spectra of aqueous solution of MO (0.1 mM) in presence of Ag_2_Te (0.005 g/L) at different irradiation times. It can be observed from graphs that the maximum absorbance at 510 nm decreased and finally disappeared after 60 min representing the complete degradation of dye. On the other hand, when the same experiment was done in the lack of Ag_2_Te nano-powder, solitary 9% degradation was seen constant after irradiation for 6 h. In the presence of Ag_2_Te nanopowder and absence of UV light, 19% degradation was observed after 6 h. It is because of the adsorption of dye on surface of Ag_2_Te. According to these experiments the presence of both catalyst and UV light are necessary for an efficient degradation of dye from aqueous solution. When Ag_2_Te nanopowder is irradiated by light with energy greater than or equal to band-gap, in the valence band an electron could be excited to the conduction band, and generated a hole in the valance band. These electron-hole pair that generated by UV-light can also recombines or interacts independently with other molecules in aqueous solution. The holes in the valence band could be reacted with water on the surface of Ag_2_Te or hydroxide ions and produced extremely reactive hydroxyl radicals (˙OH) whereas electrons could accept oxygen by adsorption and shaped superoxide radical anion (O_2_^−^). This radical may form organic peroxides or H_2_O_2_ in presence of organic adsorbent. The hydroxyl radical is a very strong oxidizing agent and aggress the dye molecule to provide the oxidized product. The reactions could be summarized in following reactions^47^,^48^,^49^,^50^:


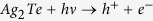



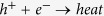



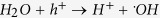



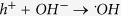



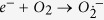



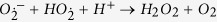



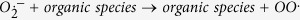



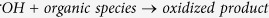


Figure 4c shows schematically reactions that were performed in presence of UV-light in aqueous solution. In these reactions the pair of electron and hole played main rule.

According to this result, Ag_2_Te can introduce as an effective photo-catalytic agent in aqueous solution for degradation of organic pollution in presence of UV-light.

For investigation the key factor on photo-catalytic activity of Ag_2_Te nanostructures, the photocatalytic behavior have been studied in different morphologies and different size of product. Nanoparticles and nanorod structures with two size have been applied in photo-catalytic reaction and compared the efficiency of degradation on 60 min. The Sample No. 6, 13, 14 and 19 have been used for degradation of Methyl orange in presence of UV light. The degradation of dye diagrams are shown in the Fig. 4d. The calculated percentage of degradation MO for Samples No. 6, 13, 14 and 19 are 88%, 94%, 95% and 84% respectively. According to results and morphology of product, the nanoparticles that have larger surface area show the better efficiency for degradation in comparison to nanorod structures.

Therefore one of the other key factors on degradation of dye in presence of photo-catalysts is the morphology of nanostructure. The nanoparticles because of high surface area show better degradations efficiency.

## Methods

### Materials

All the chemicals such as silver nitrate(AgNO_3_), tellurium tetrachloride (TeCl_4_), hydrazine hydrate (N_2_H_4_.H_2_O), cetyltrimethylammoniumbromide (CTAB), sodiumdodecyl sulfate (SDS), polyethyleneglycol (PEG600) and polyvinylpyrrolidone (PVP25000) were of analytical grade and were purchased from Merck Company (pro-analysis) and used without further purification. A Teflon-lined stainless steel cylindrical closed chamber with 150 ml capacity was used for the synthesis.

### Physical measurements

XRD patterns were recorded from a diffractometer of Philips Company with X’PertPro filtered by Cu Ka radiation (λ = 1.54 Å). Microscopic morphology of products was achieved by a LEO 1455VP scanning electron microscope. Prior to taking images, the samples were coated by a very thin layer of Pt to make the sample surface conductor and prevent charge accumulation, and obtaining a better contrast. FT-IR spectra were recorded on Galaxy series FTIR5000 spectrophotometer. Room temperature photoluminescence was studied by a Perkin Elmer fluorescence instrument. The UV-Vis spectra were taken on a JASCO UV–Visible scanning spectrometer (Model V-670).

### Synthesis of silver telluride nanostructures

In a typical synthesis, AgNO_3_ and TeCl_4_ were dissolved in distilled water separately and were mixed together, N_2_H_4_.H_2_O was then added drop wise to the mixed solution. After stirring, the reactants were put into a 150 ml capacity Teflon-lined autoclave. The autoclave was maintained at 160°C for 6 h and then cooled to room temperature naturally. The gray precipitate was washed with alcohol and distilled water several times and was dried in oven at 50 °C for 10 h. A series of experiments were done under the preferred conditions in order to study different factors on the morphology and purity of products (Table 1).

### Photo-degradation of methyl orange

The photo-degradation was executed in a home-made glass reactor system containing 200 mL of aqueous solution of Methyl Orange with 10 ppm concentration in pH = 2, 0.005 g/L of photo-catalyst Ag_2_Te powder. The suspension is set aside by magnetic stirring (500 rpm) at room temperature and was laid under dark conditions for 30 min. After that, the system is irradiated by UV lamp (Osram ULTRA-VITALUX 300 W).

This lamp releases a UVA mixture, ranging from 320 to 400 nm and UVB with 290–320 nm wavelengths, and it emits 13.6 and 3.0 W radiation, respectively; it is ozone-free and radiation encapsulated into a quartz tube, which is adrift into the methyl orange solution located in the center of the reactor.

## Additional Information

**How to cite this article**: Gholamrezaei, S. *et al*. Hydrothermal preparation of silver telluride nanostructures and photo-catalytic investigation in degradation of toxic dyes. *Sci. Rep.* **6**, 20060; doi: 10.1038/srep20060 (2016).

## Figures and Tables

**Table 1:** Different condition for preparation of Ag_2_Te.

S.No.	Effect	Time (h)	Temperature (°C)	Rrecursors	Reductant	Surfactant (Ratio)
1	Method	—	—	AgNO_3_ + TeCl_4_	N_2_H_4_.H_2_O	—
2	Time	6	160	AgNO_3_ + TeCl_4_	N_2_H_4_.H_2_O	—
3		12	160	AgNO_3_ + TeCl_4_	N_2_H_4_.H_2_O	—
4		18	160	AgNO_3_ + TeCl_4_	N_2_H_4_.H_2_O	—
5		24	160	AgNO_3_ + TeCl_4_	N_2_H_4_.H_2_O	—
6	Temperature	12	120	AgNO_3_ + TeCl_4_	N_2_H_4_.H_2_O	—
7		12	140	AgNO_3_ + TeCl_4_	N_2_H_4_.H_2_O	—
8		12	180	AgNO_3_ + TeCl_4_	N_2_H_4_.H_2_O	—
9		12	200	AgNO_3_ + TeCl_4_	N_2_H_4_.H_2_O	—
10	Reductant	12	120	AgNO_3_ + TeCl_4_	Na_2_SO_3_	—
11		12	120	AgNO_3_ + TeCl_4_	KBH_4_	—
12	Surfactant	12	120	AgNO_3_ + TeCl_4_	N_2_H_4_.H_2_O	CTAB (1:1)
13		12	120	AgNO_3_ + TeCl_4_	N_2_H_4_.H_2_O	CTAB (1:2)
14		12	120	AgNO_3_ + TeCl_4_	N_2_H_4_.H_2_O	SDS (1:1)
15		12	120	AgNO_3_ + TeCl_4_	N_2_H_4_.H_2_O	SDS (1:1)
'16		12	120	AgNO_3_ + TeCl_4_	N_2_H_4_.H_2_O	PVP25000 (1:1)
17		12	120	AgNO_3_ + TeCl_4_	N_2_H_4_.H_2_O	PVP25000 (1:2)
18		12	120	AgNO_3_ + TeCl_4_	N_2_H_4_.H_2_O	PEG600 (1:1)
19		12	120	AgNO_3_ + TeCl_4_	N_2_H_4_.H_2_O	PEG (1:2)
